# Supplementary material for: Unveiling the dynamic active site of defective carbon-based electrocatalysts for hydrogen peroxide production
Source: Nat Commun. 2023 Oct 7;14:6275. doi: 10.1038/s41467-023-41947-7 (PMC10560253; doi:10.1038/s41467-023-41947-7)
Supplement: Supplementary file 1 — Supplementary Information [file 41467_2023_41947_MOESM1_ESM.pdf]

Supplementary Information

Unveiling the dynamic active site of defective carbon-based electrocatalysts for hydrogen peroxide production

Qilong Wu,[1,2,3+] *Haiyuan Zou,*[4,+] *Xin Mao,*[5,+] *Jinghan He,*[1] *Yanmei Shi,*[6] *Shuangming Chen,*[7] *Xuecheng Yan,*[3] *Liyun Wu,*[1] *Chengguang Lang,*[3,8] *Bin Zhang,*[6] *Li Song,*[7] *Xin Wang,*[9,10] *Aijun Du,*[5] *Qin Li,*[3] *Yi Jia,*[9,10]* *Jun Chen*[2,]* *Xiangdong Yao*[1,8,]*

[1]State Key Laboratory of Inorganic Synthesis and Preparative Chemistry, College of Chemistry, Jilin University, Changchun 130012, P. R. China.

[2]Intelligent Polymer Research Institute, Australian Institute for Innovative Materials, Innovation Campus, University of Wollongong, Squires Way, North Wollongong, NSW 2500, Australia.

[3]School of Environmental engineering and Built Environment, Griffith University, Nathan Campus, Brisbane, QLD 4111, Australia.

[4]Guangdong Provincial Key Laboratory of Energy Materials for Electric Power, Southern University of Science and Technology, Shenzhen 518055, China.

[5]School of Chemistry and Physics and Centre for Materials Science, Queensland University of Technology, Gardens Point Campus, Brisbane, 4001, Australia.

[6]School of Science, Institute of Molecular Plus, Tianjin University, Tianjin, 300072 China

[7]Hefei National Laboratory for Physical Sciences at the Microscale, iChEM (Collaborative Innovation Center of Chemistry for Energy Materials), School of Chemistry and Materials Science, and National Synchrotron Radiation Laboratory, University of Science and Technology of China, Hefei, Anhui, 230026 P. R. China.

[8]School of Advanced Energy, Sun Yat-Sen University (Shenzhen), Shenzhen, Guangdong 518107, P. R. China.

[9]Petroleum and Chemical Industry Key Laboratory of Organic Electrochemical Synthesis, College of Chemical Engineering, and Zhejiang Moganshan Carbon Neutral Innovation Institute, Zhejiang University of Technology, 18 Chaowang Road, Gongshu District, Hangzhou 310032, P. R. China.

[10]Zhejiang Carbon Neutral Innovation Institute, Moganshan Institute ZJUT, Kangqian District, Deqing 313200, P. R. China

*Corresponding author. E-mail: jiayi@zjut.edu.cn; junc@uow.edu.au; yaoxd3@mail.sysu.edu.cn

[+]These authors contributed equally to this work.

Supplementary Figures:

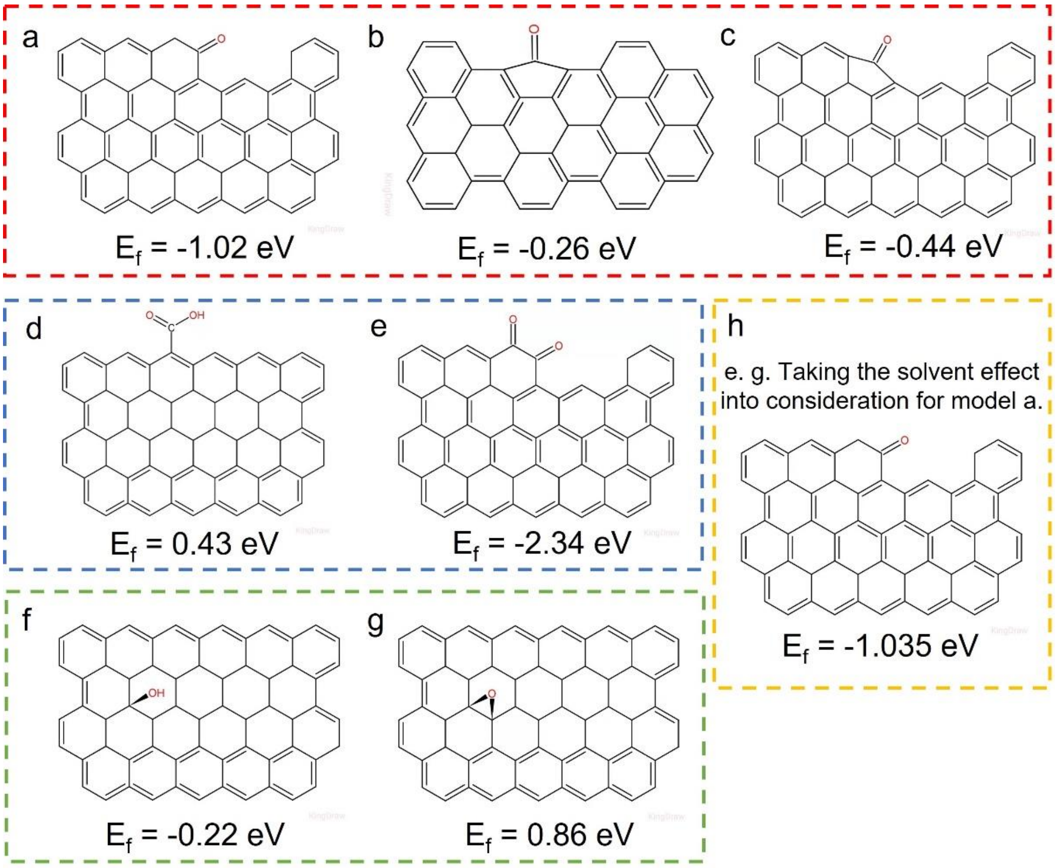

Supplementary Fig. 1 | The DFT calculated formation energy of O-groups on the different position of graphene and defective graphene. a-g, Formation energy of various O-groups on graphene and defective graphene (without taking solvent effect into consideration). h, Formation energy of model a after taking the solvent effect into consideration. For comparison, an implicit solvent effect has been calculated, and we found the formation energy only changes a little bit. The calculated formation energy for structure a is -1.035 eV, so we believe the solvent in the system have a limited impact on our calculation.

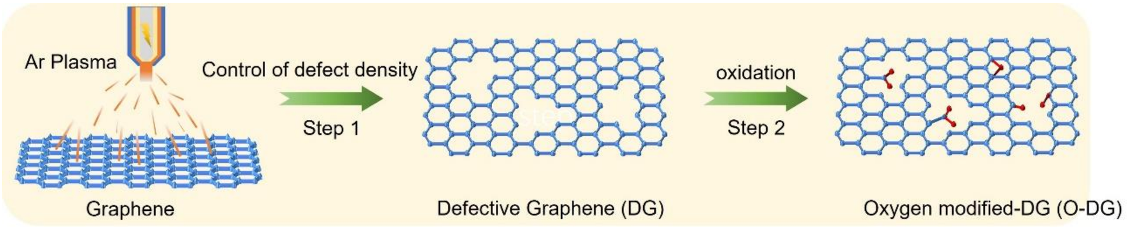

Supplementary Fig. 2 | Schematic diagram of the fabrication processes of O-DG catalysts. The Ar-plasma treatment was used to fabricate the defect sites on graphene (step 1). Then, the O-groups were modified on defective graphene using H_2O_2 oxidation method (step 2).

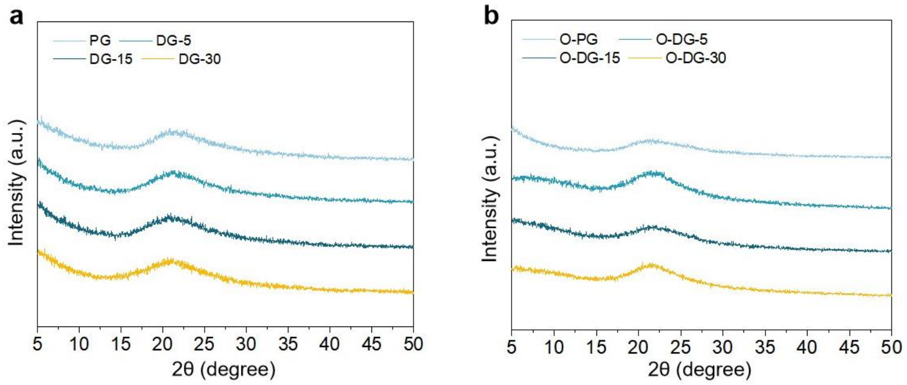

Supplementary Fig. 3 | PXRD patterns of samples. a, PXRD patterns of PG, DG-5, DG-15 and DG-30. b, PXRD patterns of O-PG, O-DG-5, O-DG-15 and O-DG-30.

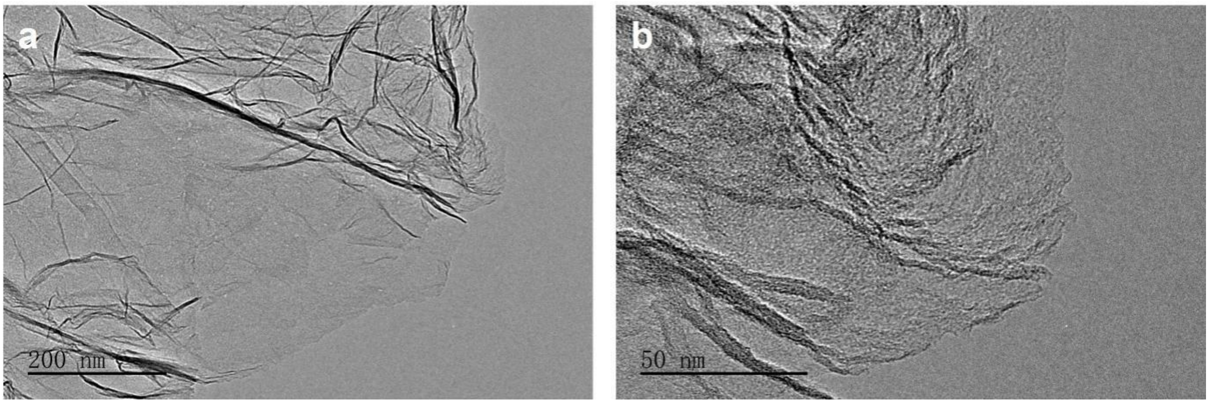

Supplementary Fig. 4 | Structural characterization of O-PG. a, b TEM images of O-PG.

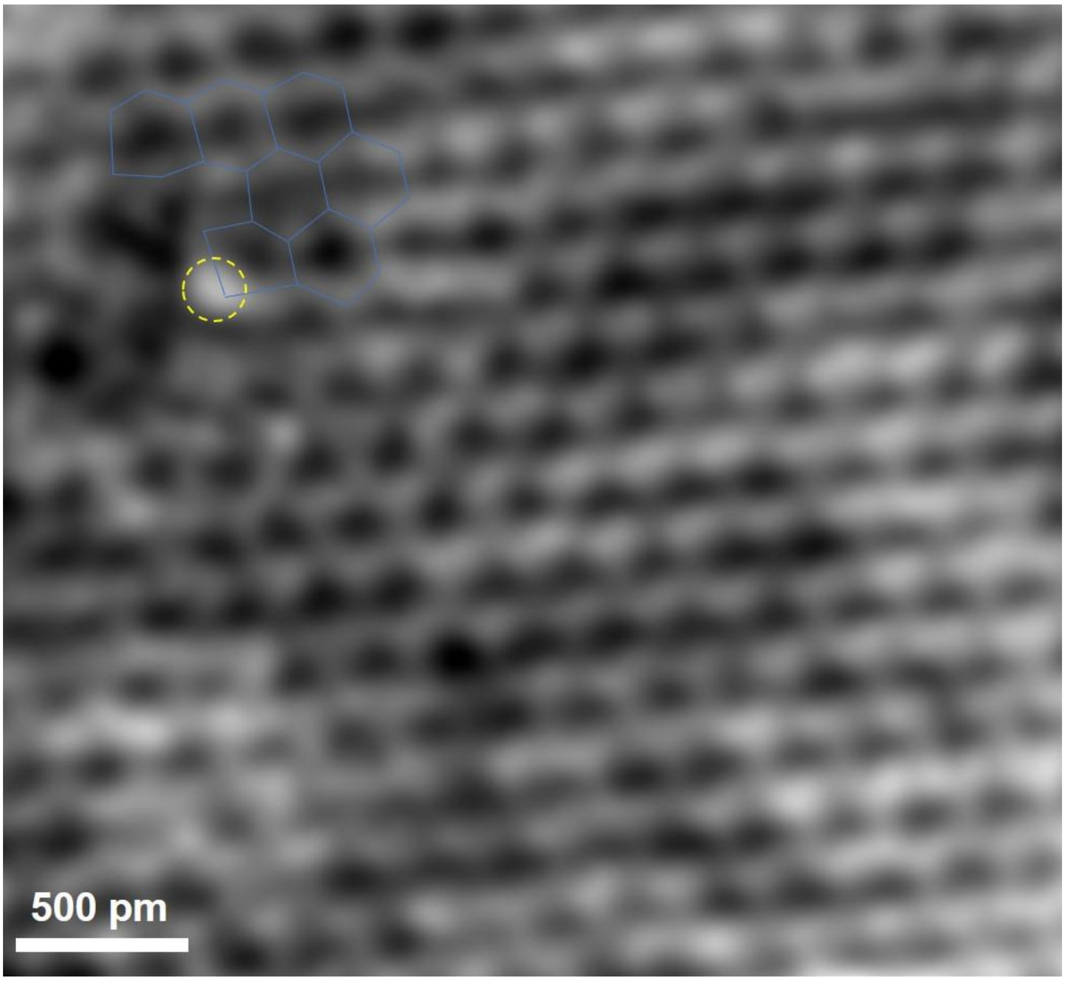

Supplementary Fig. 5 | iDPC-STEM image of O-DG-30 with partial defective domain and major perfect domain.

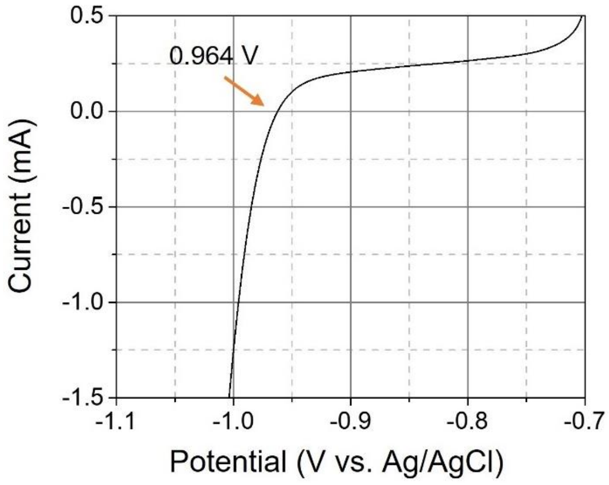

Supplementary Fig. 6 | Calibration of reference electrodes and conversion to RHE. The calibration of Ag/AgCl electrodes was performed in a three-electrode system (working electrode: polished Pt mesh, counter electrode: Pt mesh, reference electrode: Ag/AgCl electrode). High purity H_2 was used to pre-purge and saturate the electrolytes. Linear scanning voltammetry (LSV) was run at a scan rate of 1 mV s^{-1}, and the potential at which the current crossed zero is taken to be the thermodynamic potential for the hydrogen electrode reactions (As shown in above). For example, in 0.1 M KOH electrolyte, the zero current point is at -0.964V, so E (RHE) = EAg/AgCl + $E^{\theta}_{Ag/AgCl}$ + 0.964 V.

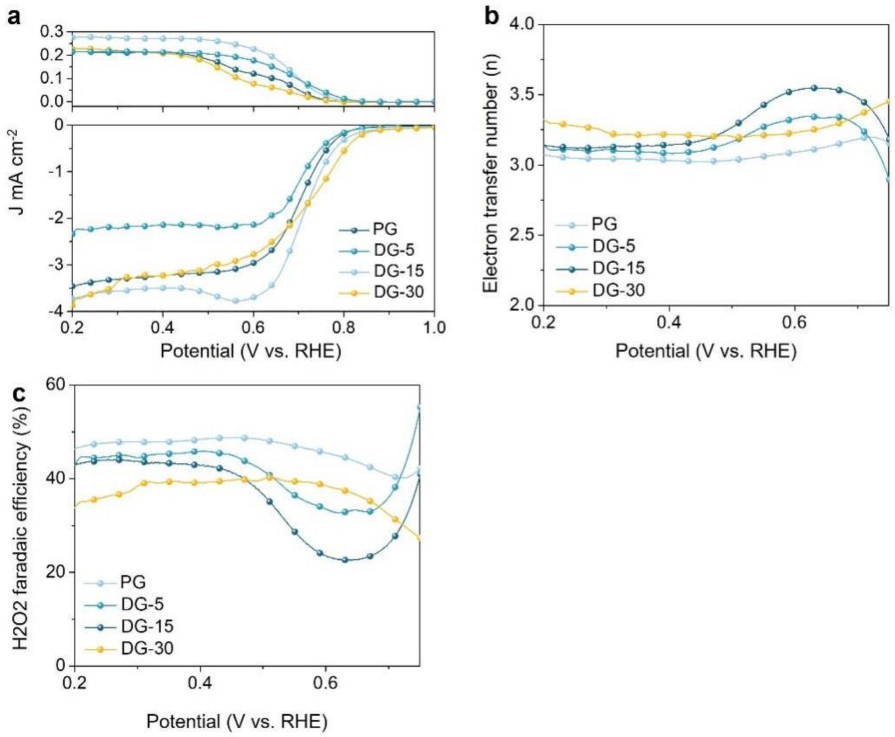

Supplementary Fig. 7 | Electrocatalytic performacne of PG and DG samples. a, The polarization curves of PG, DG-5, DG-15, and DG-30 on RRDE at 1600 rpm in O_2 -saturated 0.1 M KOH (scan rate: 10 mV s^{-1}). b, The electron transfer number of PG, DG-5, DG-15, and DG-30. c, The H_2O_2 faradaic efficiencies of PG, DG-5, DG-15, and DG-30. (resistance in RRDE test system: 21.3±2.4, iR compensation rate: 95%)

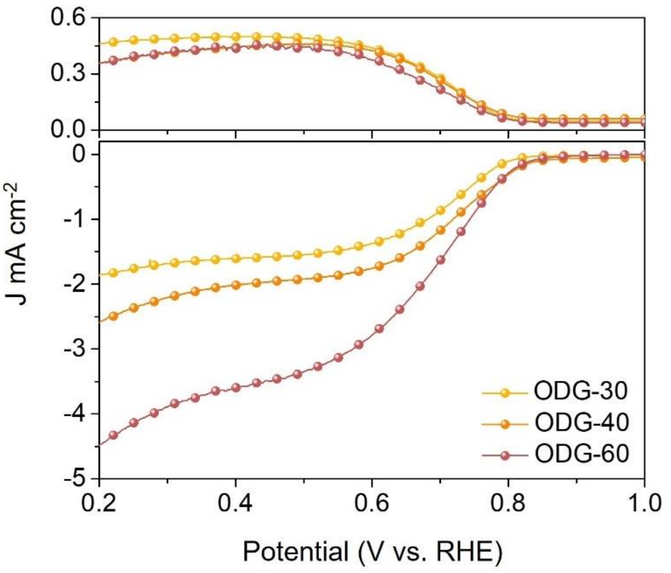

Supplementary Fig. 8 | The polarization curves of O-DG-30, O-DG-40, and O-DG-60 on RRDE at 1600 rpm in O_2-saturated 0.1 M KOH. (scan rate: 10 mV s^{-1}, resistance in RRDE test system: 21.3±2.4, iR compensation rate: 95%).

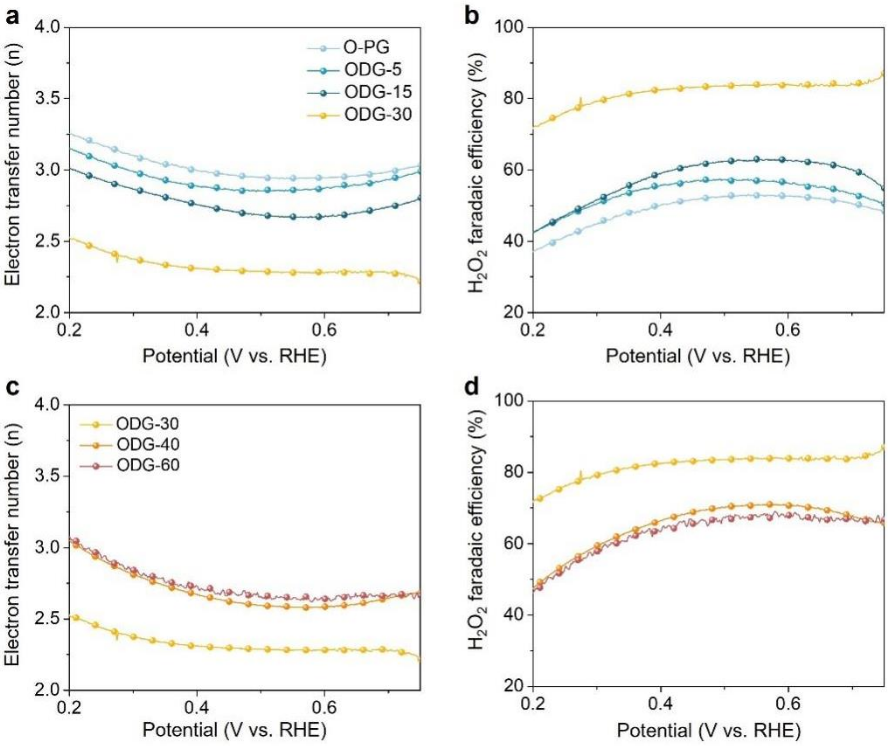

Supplementary Fig. 9 | Electrocatalytic performacne of O-PG and O-DG samples.a, The electron transfer number of O-PG, O-DG-5, O-DG-15, and O-DG-30. b, The H_2O_2 faradaic efficiencies of O-PG, O-DG-5, O-DG-15, and O-DG-30. c, The electron transfer number of O-DG-30, O-DG-40, and O-DG-60. d, The H_2O_2 faradaic efficiencies of O-DG-30, O-DG-40, and O-DG-60. (resistance in RRDE test system: 21.3±2.4, iR compensation rate: 95%)

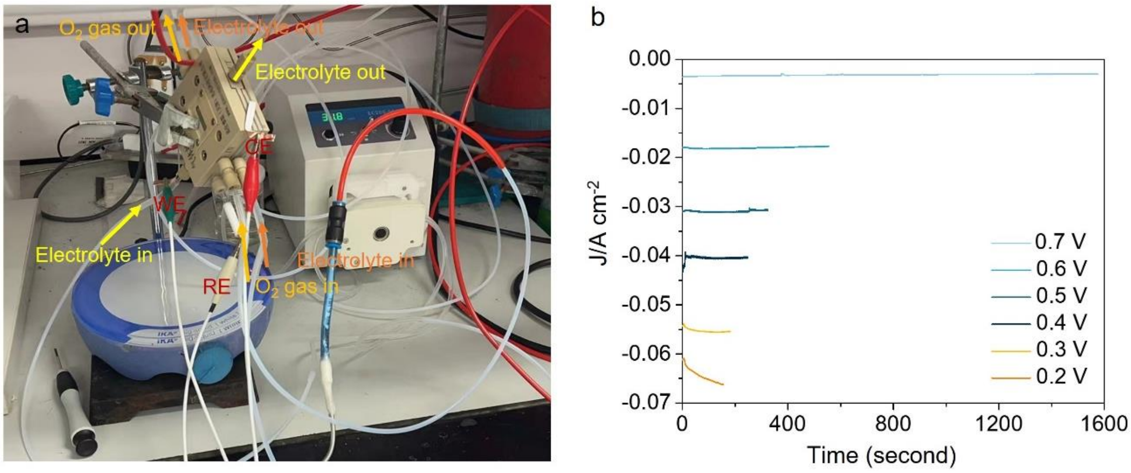

Supplementary Fig. 10 | 2 e^- ORR performance evaluation of O-DG-30 catalyst in the flow cell test system. a, The photograph of flow cell test system (WE: working electrode, CE: counter electrode, RE: reference electrode). b, The I-T curves of O-DG-30 at various potentials. (quantity of electricity: 5C for 0.7 V vs. RHE and 10C for other potentials, resistance in flow cell test system: 7.2±1.6, iR compensation rate: 95%)

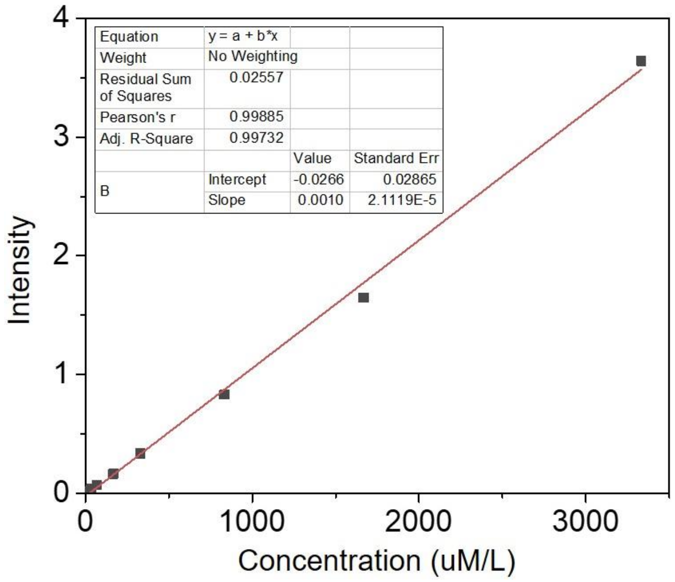

Supplementary Fig. 11 | The calibration curve of H_2O_2 using the method of potassium titanium (IV) oxalate by UV-Vis spectrophotometer at a wavelength of 400 nm.

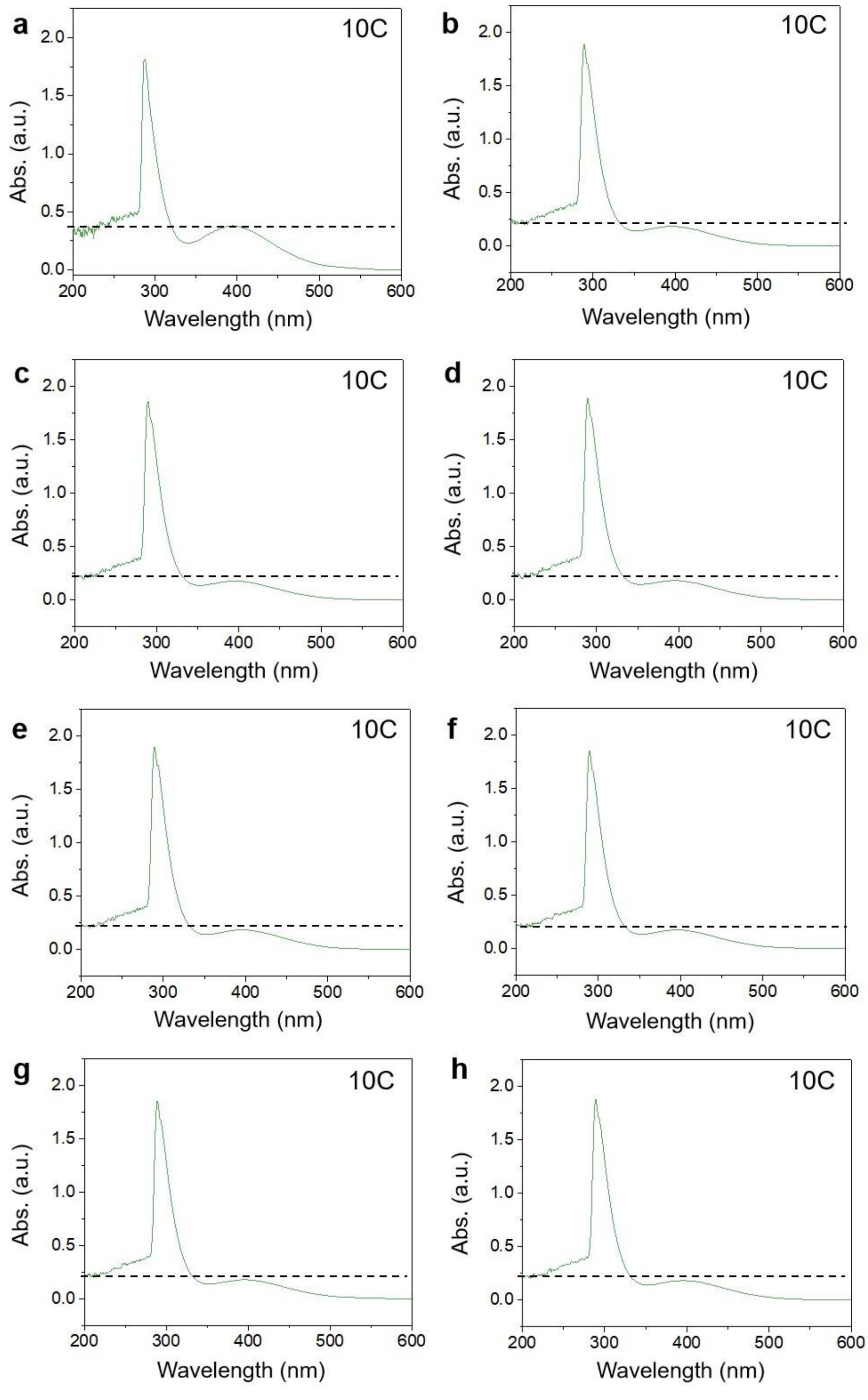

Supplementary Fig. 12 | The 8 cycling test of O-DG-30 in flow cell under intermittent working mode. a-h, The UV-vis adsorption curves of electrolyte after I-T test.

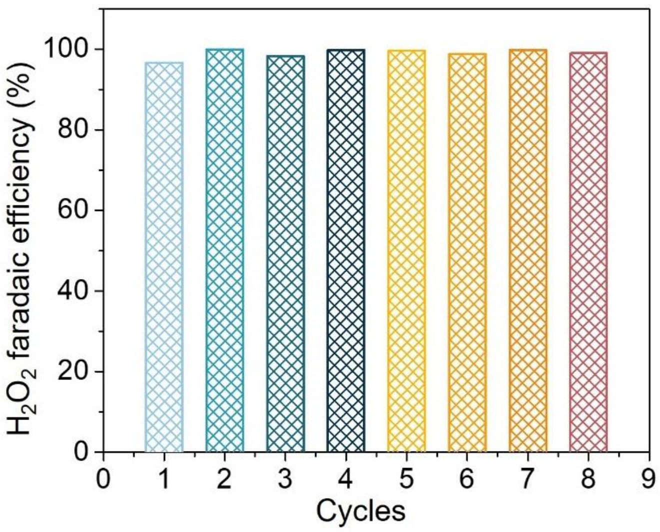

Supplementary Fig. 13 | The Faradaic efficiencies of O-DG-30 were tested by a flow cell under intermittent working conditions.

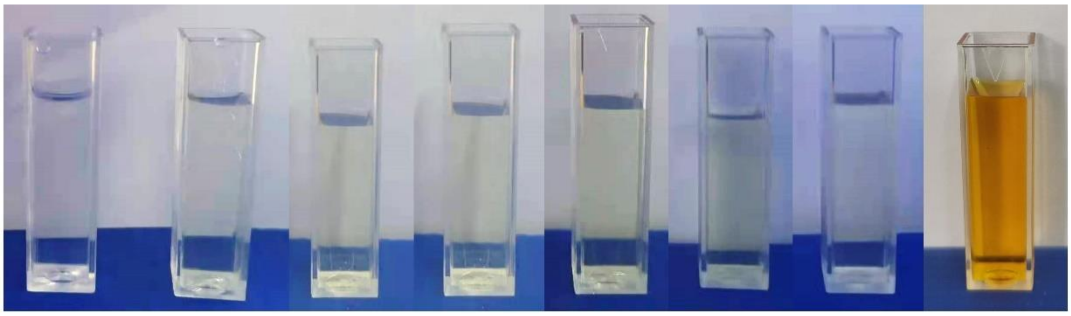

Supplementary Fig. 14 | Images of the electrolyte after chromogenic reaction using the method of potassium titanium (IV) oxalate. (quantity of electricity: 5C for 0.7 V vs. RHE and 10C for other potentials, 10 hours continuously tested at 0.55 V vs. RHE).

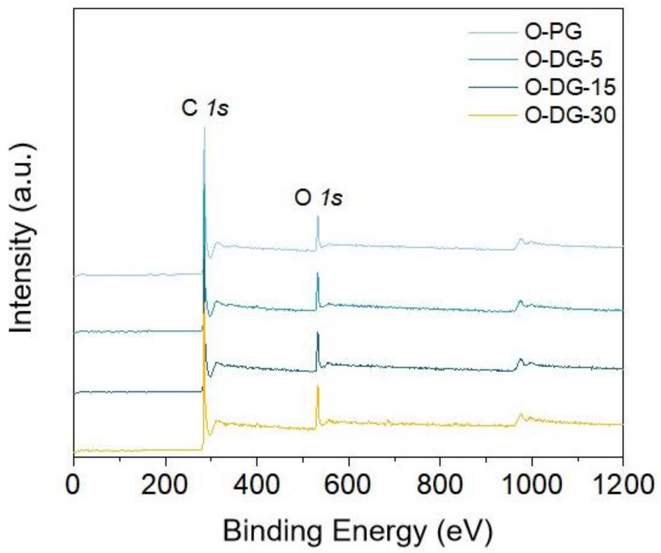

Supplementary Fig. 15 | XPS survey scan of O-PG, O-DG-5, O-DG-15, O-DG-30. The XPS survey scan of samples shows obvious O 1s signals, demonstrated that O group was successfully modified on the defective graphene.

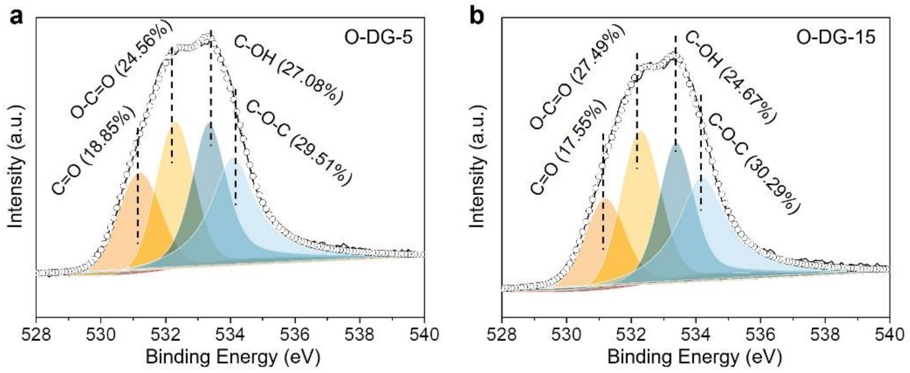

Supplementary Fig. 16 | XPS O *1s* spectra of amples. a, O *1s* spectra of O-DG-5 and b, O *1s* spectra of O-DG-15. Further conbined with the fig. 3e and 3f, the variation trend and relationship between O group types and carbon defect density was summarized in Supplementary Table 1.

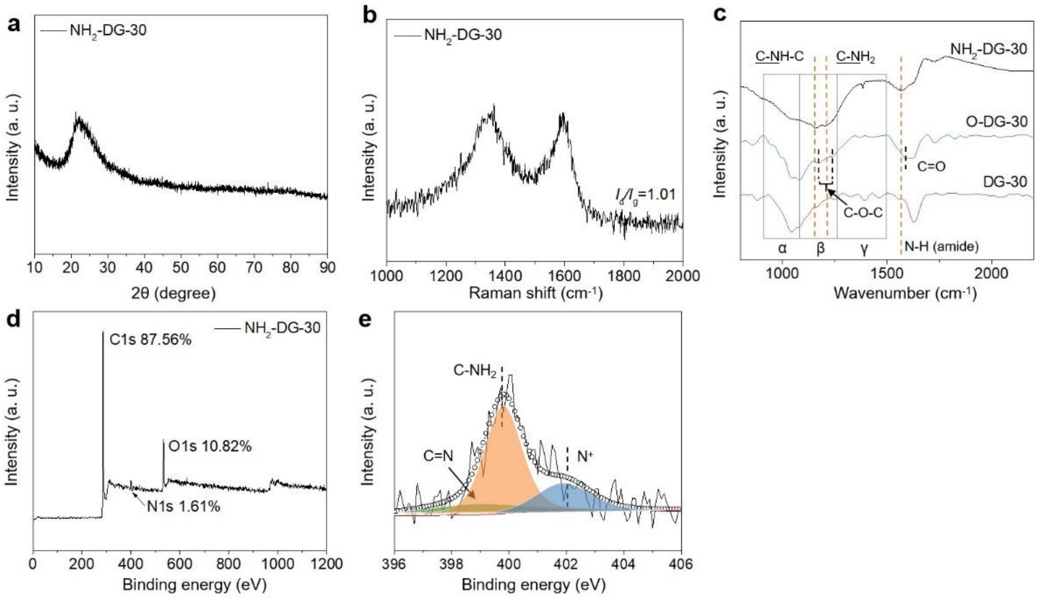

Supplementary Fig. 17 | Characterization of NH_2-DG-30. a, XRD pattern of NH_2-DG-30. b, Raman spectrum of NH_2-DG-30. c, FTIR spectra of NH_2-DG-30. d,e, XPS patterns (survey scan and N*1s* spectrum) of NH_2-DG-30.

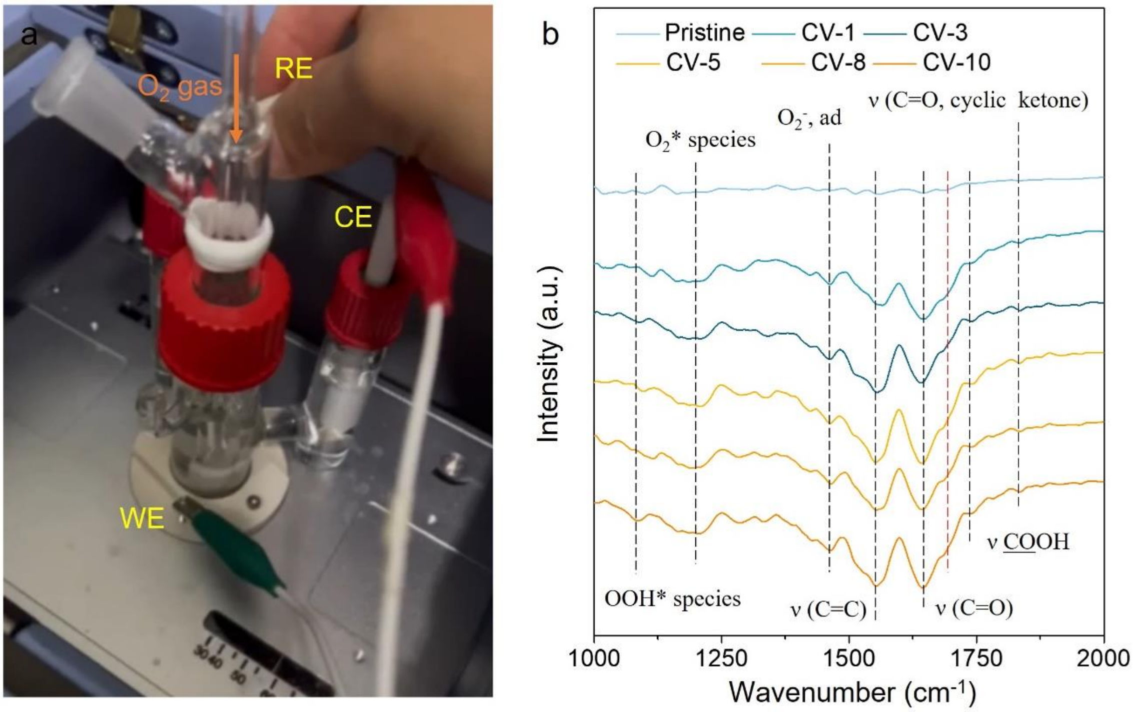

Supplementary Fig. 18 | The i*n-situ* attenuated total reflectance infrared (ATR-IR) electrochemical test for O-DG-30 catalyst. a, The photograph of ATR-IR electrochemical cell (WE: working electrode, CE: counter electrode, RE: reference electrode). b, The ATR-IR spectra of O-DG-30 after different CV cycles activation.

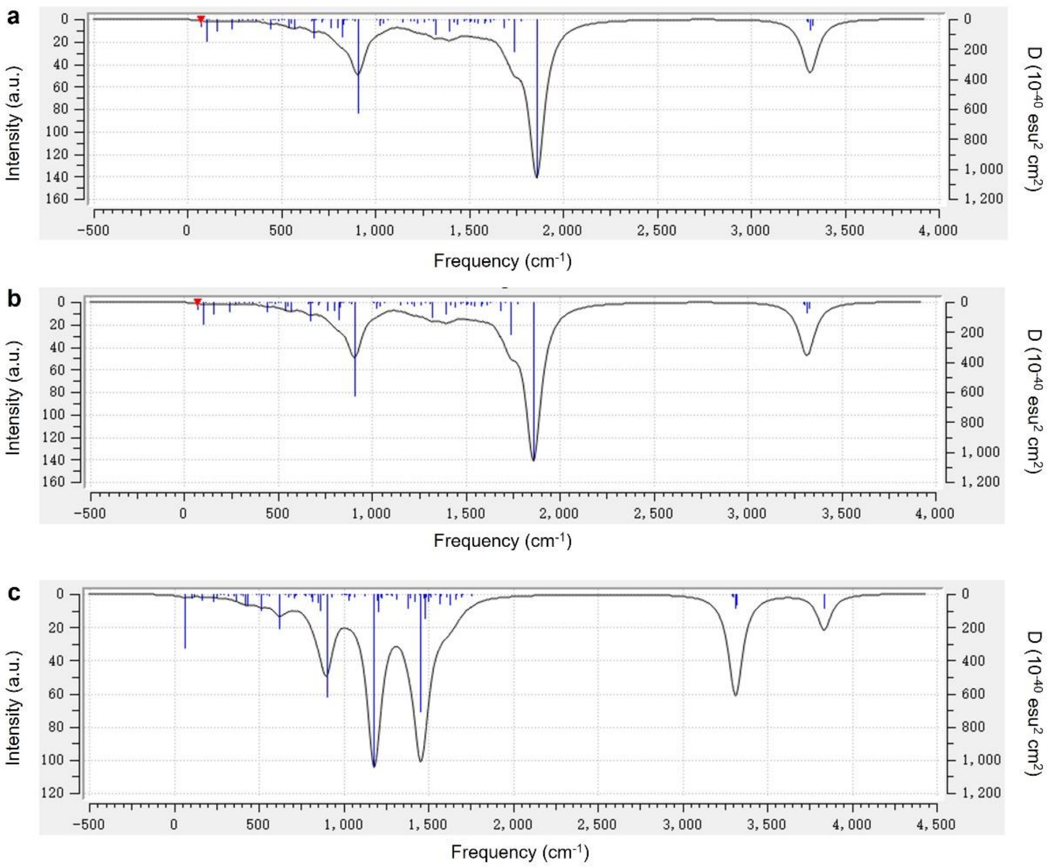

Supplementary Fig. 19 | The original calculated IR spectra of three possible atomic structures of the O-groups and relevant surface species on defective graphene, including a) pentagon=O; b) pentagon=O+OOH*; c) pentagon=OH.

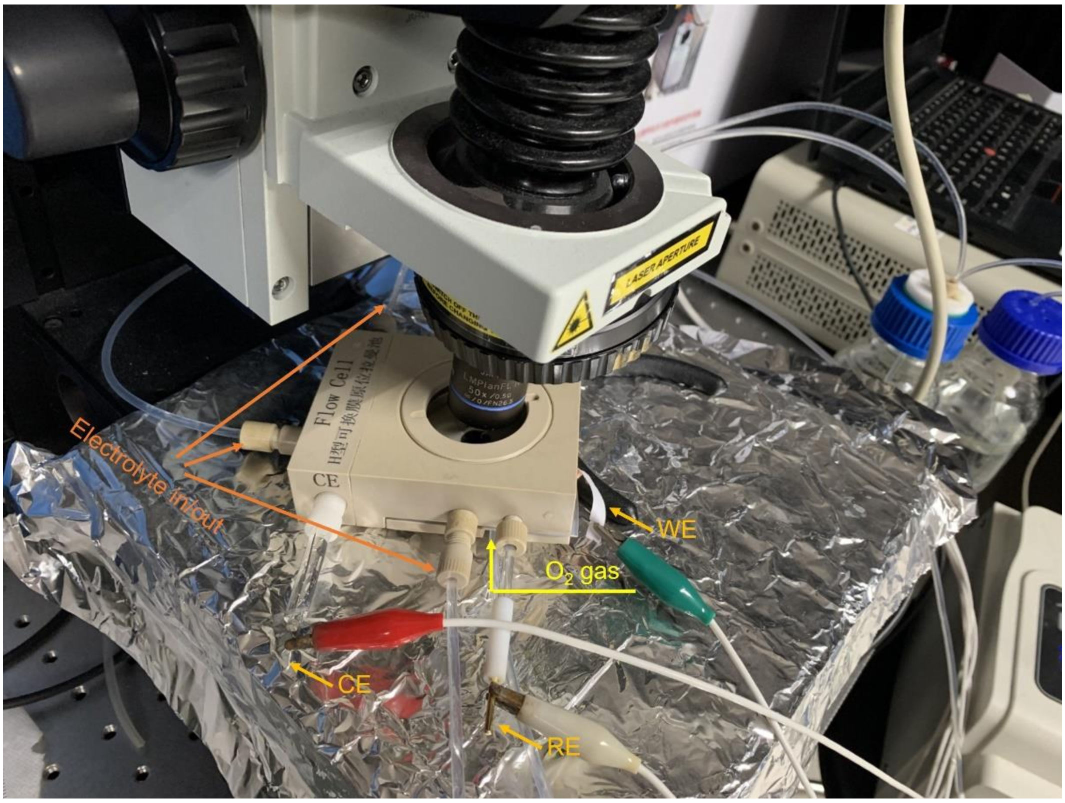

Supplementary Fig. 20 | The photograph of the flow type Raman cell (WE: working electrode, CE: counter electrode, RE: reference electrode).

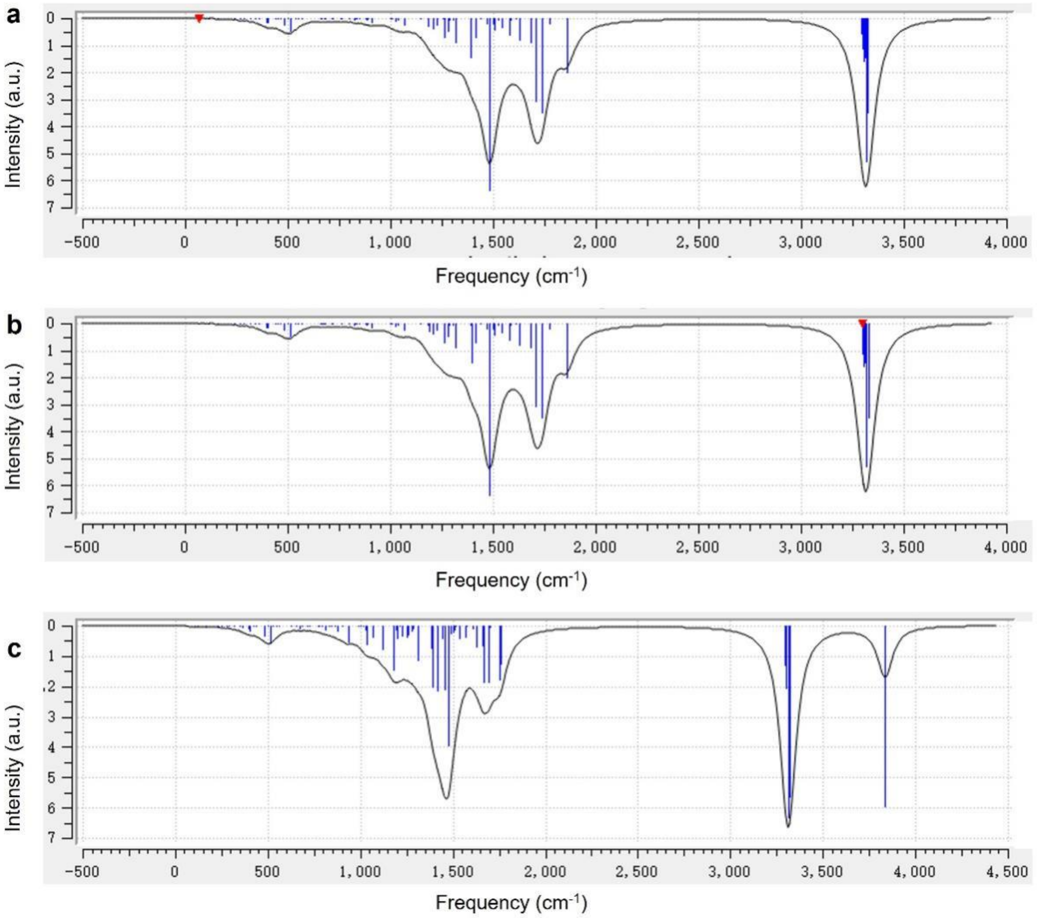

Supplementary Fig. 21 | The original calculated Raman spectra of three possible atomic structures of the O-groups and relevant surface species on defective graphene, including a) pentagon=O; b) pentagon=O+OOH*; c) pentagon=OH.

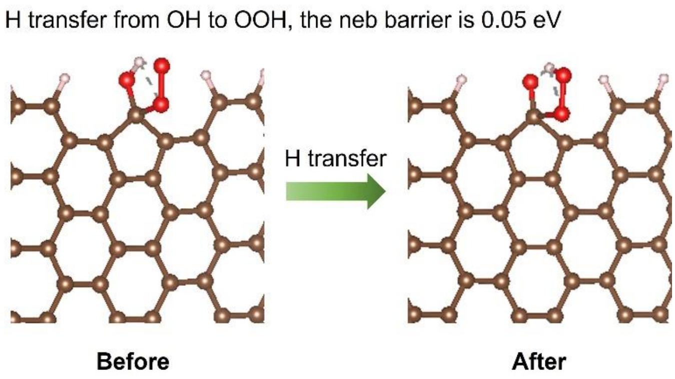

Supplementary Fig. 22 | The calculated net barrier of H transfer from pentagon=OH+O_2^-* to pentagon=O+OOH* (brown: C atom; red: O atom; white: H atom).

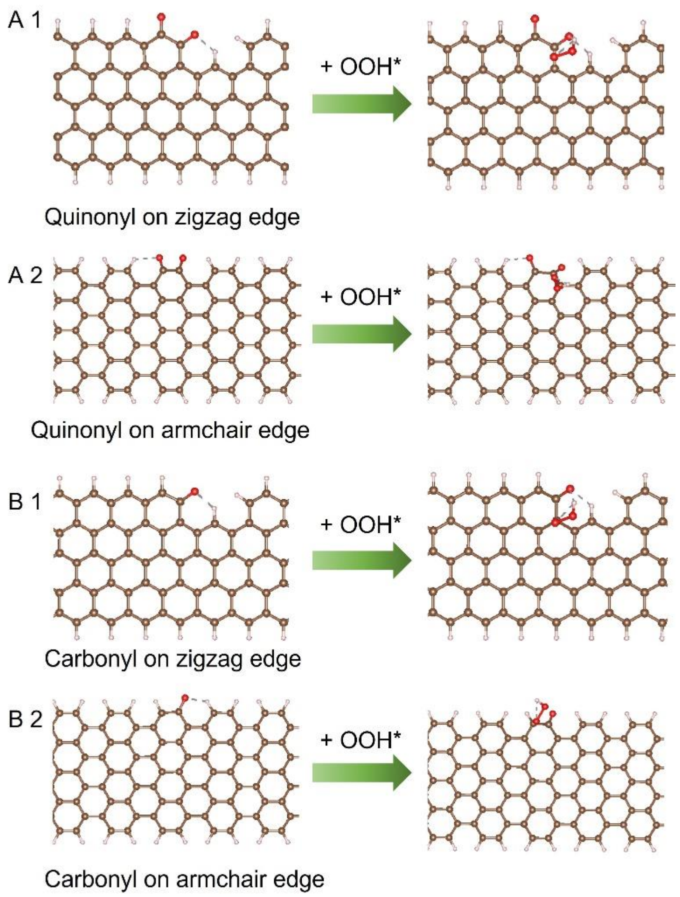

Supplementary Fig. 23 | The atomic structures of the examined O-groups on edge defect sites (left) and the atomic structures of models after the first proton electron stransfer step (right).

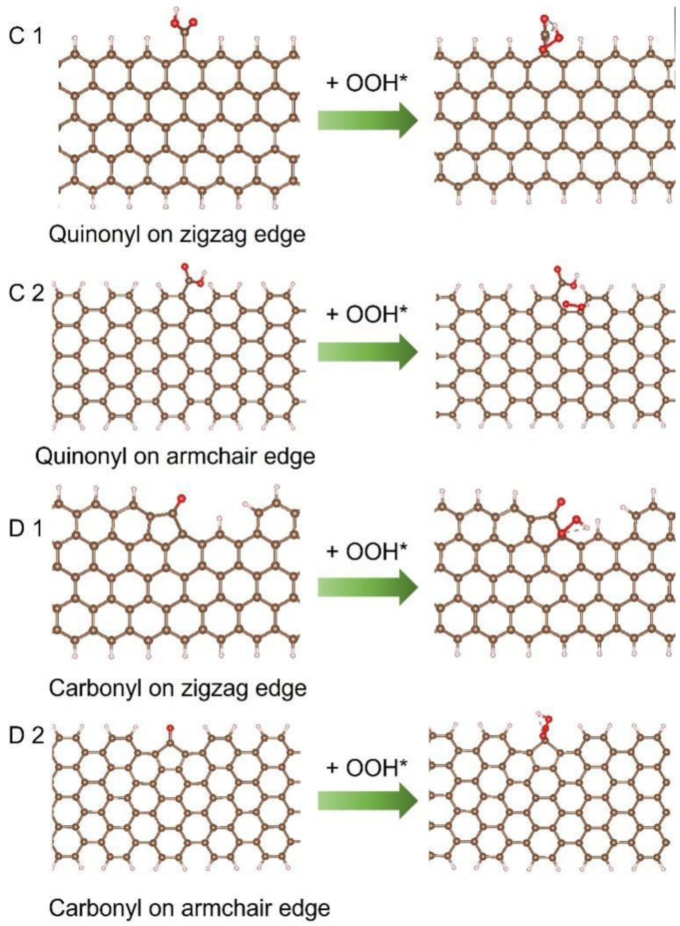

Supplementary Fig. 24 | The atomic structures of the examined O-groups on edge and pentagon defect sites (left) and the atomic structures of models after the first proton electron stransfer step (right).

Supplementary Table 1 | The variation of O-group types with the increase of Ar-plasma treatment time.

	C=O (%)	O-C=O (%)	C-OH (%)	C-O-C (%)
O-PG	13.68	22.79	38.80	24.71
O-DG-5	18.85	24.56	27.08	29.51
O-DG-15	17.55	27.49	24.67	30.29
O-DG-30	23.95	30.37	21.86	23.86

Supplementary Table 2 | The CV-dependent C/O ratios of O-DG-30 during electrocatalysis.

	C (%)	O (%)
CV-1	81.40	18.60
CV-3	81.79	18.21
CV-6	83.77	16.32
CV-10	82.26	17.74

Supplementary Table 3 | The variation of O-group types of DG-30 at different CV cycles activation.

	C=O (%)	O-C=O (%)	C-OH (%)	C-O-C (%)
Pristine	23.95	30.37	21.86	22.83
CV-1	33.17	39.31	16.97	10.55
CV-6	38.80	34.26	16.98	9.91
CV-10	43.86	27.20	17.85	11.10

Supplementary Table 4 | The comparison of current density and the corresponding H2O2 selectivity between the O-DG-30 with previously reported 2e⁻ ORR catalysts was conducted under a flow cell test system.

Catalysts	current density (mA cm^{-2})	Corresponding H_2O_2 selectivity	Ref.
O-DG-30	76.6	98.38%	This work
OCN-S900	50	60-80%	[1]
Co−N SAC_{Dp}	50	~72%	[2]
N-CNMC-500	~38	~70	[3]
Sb-NSCF	~50	94.7	[4]
c-Mo/NCPs	~45	~80	[5]
PTFE-CB	60	~82	[6]

Supplementary reference

1. Chen, S., et al. Chemical Identification of Catalytically Active Sites on Oxygen-doped Carbon Nanosheet to Decipher the High Activity for Electro-synthesis Hydrogen Peroxide. *Angew. Chem., Int. Ed.*, **60**, 16607-16614 (2021).

2. Chen S, Luo T, Li X, et al. Identification of the Highly Active Co-N_4 Coordination Motif for Selective Oxygen Reduction to Hydrogen Peroxide. *J. Am. Chem. Soc.*, **144**, 14505-14516 (2022).

3. Bao Z, Zhao J, Zhang S, et al. Synergistic effect of doped nitrogen and oxygen-containing functional groups on electrochemical synthesis of hydrogen peroxide. *J. Mater. Chem. A.*, **10**, 4749-4757 (2022).

4. Yan M, Wei Z, Gong Z, et al. Sb_2S_3-templated synthesis of sulfur-doped Sb-NC with hierarchical architecture and high metal loading for H_2O_2 electrosynthesis. *Nat. Commun.*, **14**, 368 (2023).

5. Jin M, Liu S, Meng G, et al. Low-Coordinated Mo Clusters for High-Efficiency Electrocatalytic Hydrogen Peroxide Production. *Adv. Mater. Inter.*, **10**, 2201144 (2023).

6. Zhang, Q., Zhou, M., Ren, G., Li, Y., Li, Y., & Du, X. Highly efficient electrosynthesis of hydrogen peroxide on a superhydrophobic three-phase interface by natural air diffusion. *Nat. commun.* **11**, 1731 (2020).
